# Regulation of co-translational mRNA decay by PAP and DXO1 in Arabidopsis

**DOI:** 10.1186/s12870-025-06195-5

**Published:** 2025-02-18

**Authors:** Marie-Christine Carpentier, Anne-Elodie Receveur, Adrien Cadoudal, Rémy Merret

**Affiliations:** 1 CNRS-LGDP UMR 5096, 58 avenue Paul Alduy, Perpignan, 66860 France; 2 Université de Perpignan, Via Domitia, LGDP-UMR5096, 58 avenue Paul Alduy, Perpignan, 66860 France; 3 Institut de biologie moléculaire des plantes, CNRS, Université de Strasbourg, Strasbourg, France

**Keywords:** Arabidopsis, Co-translational mRNA decay, XRN4, DXO1, FRY1, 3ʹ-phosphoadenosine 5ʹ-phosphate

## Abstract

**Background:**

mRNA decay is central in the regulation of mRNA homeostasis in the cell. The recent discovery of a co-translational mRNA decay pathway (also called CTRD) has changed our understanding of the mRNA decay process. This pathway has emerged as an evolutionarily conversed mechanism essential for specific physiological processes in eukaryotes, especially in plants. In Arabidopsis, this pathway is targeted mainly by the exoribonuclease XRN4. However, the details of the molecular regulation of this pathway are still unclear.

**Results:**

In this study, we first tested the role of the 3ʹ-phosphoadenosine 5ʹ-phosphate (PAP), an inhibitor of exoribonucleases in the regulation of CTRD. Using 5’Pseq approach, we discovered that FRY1 inactivation impaired XRN4-CTRD activity. Based on this finding, we demonstrated that exogenous PAP treatment stabilizes CTRD mRNA targets. Furthermore, we also tested the implication of the exoribonuclease DXO1 in CTRD regulation. We found that DXO1, another exoribonuclease sensitive to PAP, is also involved in the CTRD pathway, probably by targeting NAD^+^-capped mRNAs. DXO1 specifically targets mRNAs linked to stress response.

**Conclusions:**

Our study provides further insights into the regulation of CTRD in Arabidopsis and demonstrates that other exoribonucleases can be implicated in this pathway.

**Supplementary Information:**

The online version contains supplementary material available at 10.1186/s12870-025-06195-5.

## Background

The tight regulation of messenger RNA (mRNA) abundance is necessary to ensure correct development and stress response in eukaryotic organisms. Changes in mRNA abundance can be induced not only by modulating the transcription level but also by modulating the mRNA decay rate. In eukaryotes, mRNA decay can occur from both extremities of the transcript. The 3’ to 5’ mRNA decay is targeted by the RNA exosome complex or SOV (suppressor of varicose) in Arabidopsis [1, 2]. The 5’ to 3’ mRNA decay takes place after the deadenylation and decapping processes. The uncapped mRNA is then targeted by an exoribonuclease (XRN1 in *Saccharomyces cerevisiae*, XRN4 in *Arabidopsis thaliana*) [3, 4].

For many years, it was proposed that mRNA decay only occurs after the last round of translation. However, several studies have suggested that translated mRNAs can also be targeted for degradation [5]. The discovery of the 5’-3’ co-translational mRNA decay (also called CTRD) validated this hypothesis. Pioneering evidence was demonstrated in yeast where uncapped mRNAs were purified from polysomal fractions [6]. This work was then followed by several other studies with the development of Next-Generation Sequencing (NGS) strategies based on the capture of 5’ monophosphate decay intermediates (also called 5’Pseq) [7–12]. These approaches revealed that mRNA decay intermediates follow a three-nucleotide periodicity. This periodicity is explained by the tracking of the last translating ribosome by the exoribonuclease. The mRNA decay rate is determined by the ribosome translocation rate codon per codon. Thus, it was proposed that in addition to mRNA decay analysis, these 5’Pseq approaches can also reveal ribosome dynamics at nucleotide resolution [8, 11–17]. The common feature identified in all 5’Pseq datasets is a 5’P reads accumulation 16 to 17 nucleotides upstream of the stop codons, explained by the slow translation termination step [11]. The dynamic of this accumulation is also used as a proxy of CTRD activity [10, 18–25].

This pathway appears to be evolutionarily conserved as it has been described in many eukaryotic and prokaryotic organisms such as bacteria, yeast, human and plants [11, 16, 19, 21, 22, 24–26]. In addition, this pathway was demonstrated to be essential for several physiological contexts. In mammalian cells, CTRD is involved in the regulation of tubulin mRNA to maintain the abundance of soluble tubulin in the cell [27–29]. In yeast, this pathway has been shown to be important for the osmotic stress response [30]. In plants, this pathway is present in at least 10 angiosperms with conserved features [19] and is regulated throughout Arabidopsis seedling development [21]. During heat stress, CTRD is also activated and has been proposed to be essential for thermotolerance [31–33]. In tomato, CTRD allows the regulation of circadian rhythm transcripts [22]. Recently, the contribution of this pathway in the general mRNA turnover was also determined in Arabidopsis, revealing that this pathway is a major determinant in mRNA turnover in both shoot and root [20]. Factors involved in the regulation of CTRD have also been described in plants. The RNA-binding protein LARP1 (La-Related protein 1) is involved in the targeting of XRN4 to polysomes especially during heat stress condition [31]. CBP80, a large subunit of the cap binding complex is also involved in the regulation of CTRD [24]. More recently, Pelota and Hbs1, two translation-related ribosome rescue factors, were proposed to be suppressors of CTRD in Arabidopsis [18]. However, details about the molecular mechanisms that trigger CTRD are still rare in the literature.

XRN family members were first described in yeast and were found to have two members: ScXRN1 and ScXRN2/RAT1 [34, 35]. Orthologs of XRN1 and XRN2 have now been identified in many organisms including Arabidopsis [36]. The Arabidopsis genome lacks an XRN1 homolog but codes for three XRNs, AtXRN2, AtXRN3 and AtXRN4, which are structurally similar to ScXRN2/RAT1 [36]. AtXRN4 which lost the bipartite NLS, found on other XRN2 homologs, during duplication functions as a cytoplasmic exoribonuclease [36]. XRN1 and XRN2 orthologs are widely conserved in eukaryotes and share two highly conserved regions called CR1 and CR2 in their N-terminal segments [37]. Recently, a crystal structure of ScXRN1 in association with the 80 S ribosome provided novel insights into the CTRD mechanism [38]. Binding to the ribosome leads to a large rearrangement of ScXRN1 structure with three flexible loops in the exoribonuclease interacting directly with the ribosome. The most prominent rearrangement takes place in loop L3 located at the end part of the CR1 domain [38]. This loop interacts with the mRNA emerging from the ribosome and is essential for the binding of ScXRN1 to ribosomes but is not essential for its catalytic activity. This loop was demonstrated to be conserved in Arabidopsis and essential for CTRD regulation [20]. However, how XRN1/XRN4 association with the ribosome is controlled remains unknown.

XRN exoribonuclease activity can be inhibited by the secondary metabolite, 3ʹ-phosphoadenosine 5ʹ-phosphate (PAP), an intermediate of the sulfate assimilation pathway and a chloroplast retrograde signal accumulating during oxidative stress in plants [39–42]. Under unstressed conditions, PAP is enzymatically degraded by the FRY1/SAL1 phosphatase (HAL2 in yeast) to form 5’AMP and Pi. In Arabidopsis, FRY1 loss-of-function leads to a constitutive overaccumulation of PAP that induces XRNs inhibition [39, 41, 42]. Through grafting experiments, it was also proposed that the FRY1-PAP retrograde pathway could play a role in long distance signaling from shoot to root to modulate XRN activities in root [42]. However, the detailed mechanism of this inhibition and the consequence on 5’-3’ mRNA turnover are unclear precisely regarding CTRD. Since FRY1 is functionally conserved, this would appear to be a conserved signaling pathway controlling XRN activity.

In addition to XRN exoribonuclease activity, PAP was also proposed to control DXO1 activity [43]. DXO proteins are involved in 5’-end quality control and possess a deNADding activity [44, 45]. These proteins also present strong deNADding activity on mRNAs with non-canonical NAD^+^ cap that consists of nicotinamide adenine dinucleotide [46]. In Arabidopsis, DXO1 activity was recently characterized. AtDXO1 crystal structure reveals conversed catalytic site but also plant-specific features [43]. Two key amino acids, E394 and D396, were demonstrated to be involved in DXO1 exoribonuclease activity. Interestingly, catalytic activity does not contribute to several phenotypes observed in a *dxo1* knockout mutant [43]. These phenotypes are linked to DXO1 N-terminal region, which is involved in the interaction with an RNA guanosine-7 methyltransferase (RNMT1) for mRNA guanosine cap methylation [47]. However, its contribution to CTRD has never been tested.

Here, we analysed the regulation of CTRD by PAP and DXO1. Using *fry1* mutant and exogenous PAP treatment, we demonstrated that PAP could affect XRN4 association with polysomes and impair CTRD activity. Using 5’Pseq approach, we demonstrated that CTRD activity is more affected in *fry1* mutant than in *xrn4* mutant suggesting the involvement of other ribonucleases in the CTRD process. Finally, we demonstrated that in addition to XRN4, DXO1 is also involved in CTRD probably by targeting NAD^+^-capped polysomal mRNAs.

## Methods

### Plant material

All Arabidopsis lines have the Col-0 background. The *xrn4-5* T-DNA line was originally ordered from the Nottingham Arabidopsis stock centre (http:/arabidopsis.info; SAIL_681_E01) and has been used in previous studies [21, 31, 32]. The *fry1-4* was previously characterized in a mutagenesis screen [39]. This mutant has a single point mutation at position 203 that generates a stop codon. This line was provided by H. Vaucheret (IJPB, Versailles, France). The DXO1(E394A/D396A) and GFP-DXO1 complemented lines were produced in [43]. The DXO1(E394A/D396A) line is expressing DXO1(E394A/D396A) fused to GFP in the *dxo1-2* (SALK_032903) background. The GFP-DXO1 complemented line is expressing DXO1 fused to GFP in the *dxo1-2* (SALK_032903) background. These two lines were provided by J. Kufel (University of Warsaw, Warsaw, Poland).

### Growth conditions

Seeds were sown on a 245 × 245 mm square plate in a single row. Seedlings were grown vertically on synthetic Murashige and Skoog medium (Duchefa) containing 1% Sucrose and 0.8% plant agar at 22 °C under a 16-h-light/8-h-dark regime. All the experiments were performed on 15-d-old seedlings. Roots and shoots were separated using a scissor at the basis of the hypocotyl and rapidly transferred to liquid nitrogen prior to RNA extraction.

### Total RNA extraction and 5’Pseq library preparation

Total RNA was isolated using Monarch Total RNA Miniprep Kit (New England Biolabs) according to manufacturer’s instructions. 5’Pseq libraries were prepared as described previously using 5 µg of total RNA as starting material [10]. After sequencing, Raw reads for Read 1 were used and trimmed to 50 pb before mapping. Metagene analysis was performed using FIVEPSEQ software with standard parameters [8]. The Terminational Stalling Index (TSI) was defined as the ratio of the number of 5’P read ends at the ribosome boundary (16–17 nt upstream from stop codons) to the mean number of 5’P read ends within the flanking 100 nt [20]. Transcripts with a TSI value higher than 3 in Col0 were used to assess CTRD activity in the different mutant backgrounds. Two distinct batches of samples were prepared. Batch 1 includes Col0, *xrn4.5* and *fry1.4* shoot and root in three independent biological replicates (18 samples, PRJNA1185437). Batch 2 includes Col0, *xrn4.5* and DXO1(E394A/D396A) in three biological replicates (18 samples, PRJNA1189285). Each batch was sequenced independently. To compare NAD^+^-capped RNAs and DXO1 targets, supplemental dataset S4 (“named NAD-RNAs identified by SPAAC-NAD-seq in m7G-depletion samples”) from [48] was used. Only transcripts identified in both datasets were kept for the analysis. To test the significance of TSI distribution, a Wilcoxson-test was performed. GO terms analysis was performed using PANTHER database using standard parameters (https://pantherdb.org).

### Polysome profile and western-blot analysis

Polysome profiles and western-blot analysis were performed as described previously [21]. Briefly, 400 mg of tissue power was homogenized in 1.2 ml of lysis buffer. After incubation 10 min on ice, lysate was clarified by centrifugation (10 min, 4 °C, 16,000 g). 900 µl of crude extract was then loaded on a 15–60% sucrose gradient. The gradient was then fractionated in 9 fractions. Fractions 1 to 3 correspond to free mRNP fractions, fraction 4 corresponds to monosome fraction and fractions 5 to 9 correspond to polysome fractions. Proteins from the different fractions were precipitated by adding 2 volumes of absolute ethanol. After 6 h at 4 °C and centrifugation, protein pellets were washed 5 times with ethanol 70%. Finally, pellets were resuspended in Laemmli 4X buffer. To analyse distribution of XRN4 and GFP-DXO1, fractions 1 to 3 were pooled to generate a free mRNA fraction and fractions 4 to 9 were pooled to generate a ribosome-bound fraction. The same percentage of each fraction was loaded and compared to an input fraction. XRN4 antibody [31] was used at 1/1,000th dilution. RPL13 antibody was purchased (Agrisera, AS13 2650) and used at 1/5,000th dilution. GFP antibody was purchased (Clontech, 632381) and used at 1/2,000th dilution. Primary antibody was incubated overnight at 4 °C under constant agitation. A horseradish peroxidase-coupled antibody was used as secondary antibody. Signal was revealed with the Immobilon-P kit (Millipore, WBULS0100).

### RT-ddPCR after transcription inhibition

In vivo transcription inhibition was performed as in [20, 49]. Plantlets were transferred horizontally in an incubation buffer (15 mM sucrose, 1 mM Pipes pH 6.25, 1 mM KCl, 1 mM sodium citrate, 1 mM cordycepin). For PAP treatment, 1 mM ATP and 1 mM PAP were added in the incubation buffer as described [50]. The time-course experiment was performed 2 h after daybreak. Roots and shoots were collected separately at 0, 30, 60 and 120 min after transcription arrest and rapidly transferred to liquid nitrogen prior to RNA extraction. 500 ng of total RNA was reverse-transcribed using SuperScript IV kit and random primers (Thermo Scientific). cDNAs were then diluted 50-fold prior to ddPCR analysis. ddPCR was performed as described previously [51]. Primers used for ddPCR are listed on Supplemental Table 1. mRNA half-life determination was performed with three independent biological replicates.

## Results

### FRY1 inactivation affects co-translational mRNA decay

**Fig. 1:**
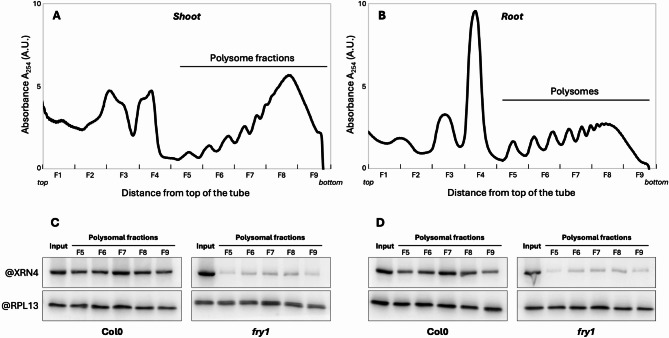
XRN4 accumulation to polysomes is impaired in *fry1*mutant. Polysomal extracts prepared from Col0 or *fry1* shoot (**A**) and root (**B**) were fractionated on a sucrose gradient. Polysome profiles were obtained by continuous 254 nm absorption measurement (A_254_ expressed in arbitrary units, A.U.). All the profiles were analysed simultaneously. Each fraction was labelled from F1 to F9. **C**-**D**. Total proteins extracted from polysomal (F5 to F9) and input fractions were analysed by immunoblotting using XRN4 or RPL13 specific antibodies (C. Shoot samples, D. Root samples). The four blots were prepared and analysed simultaneously. The same quantities of tissues were used for each condition (e.g. 300 mg of biomass)

**Fig. 2:**
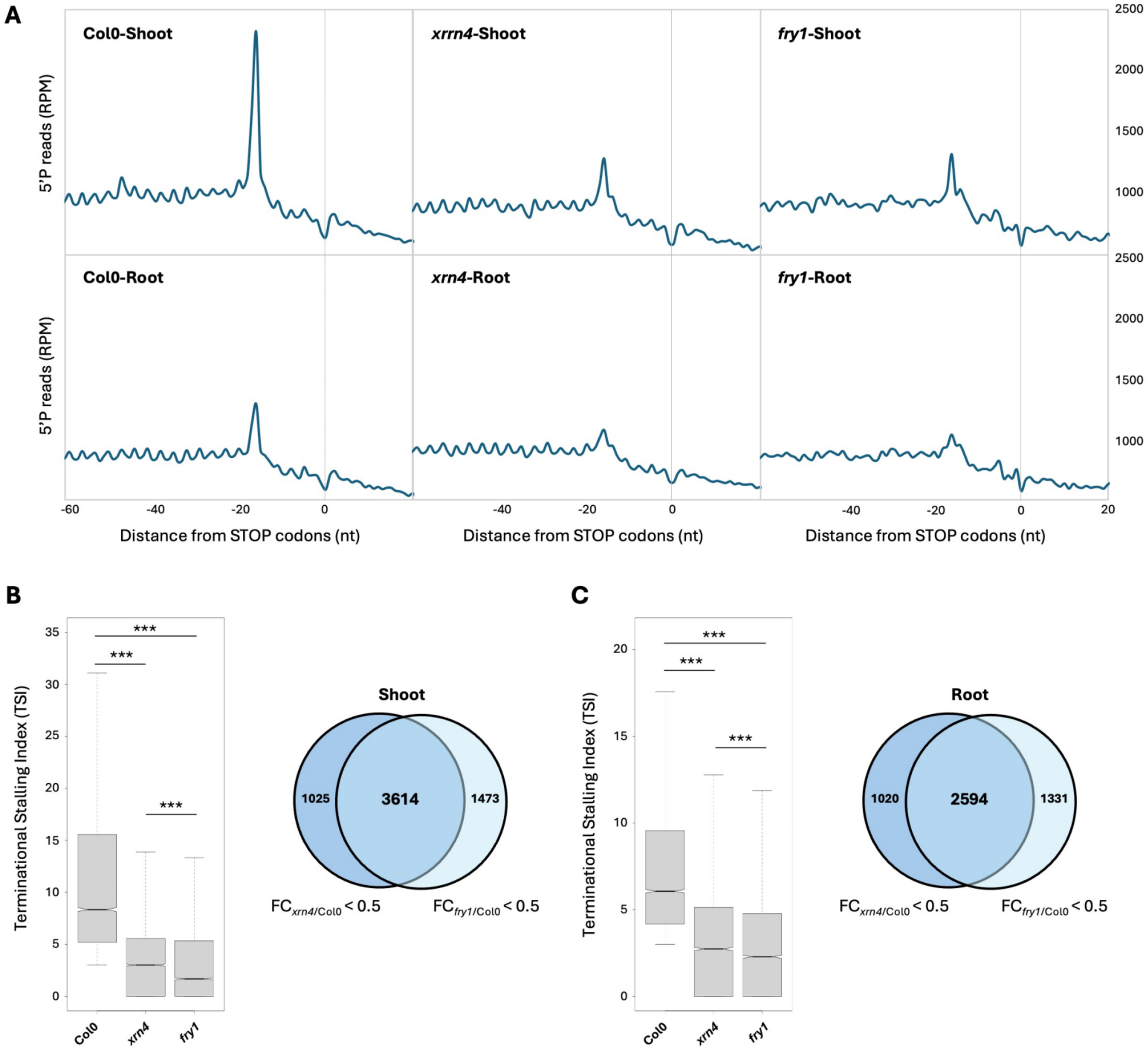
FRY1 inactivation affects co-translational mRNA decay in both shoot and root. (**A**) Metagene analysis of 5′P reads accumulation around stop codons. The different profiles are representative of 3 biological replicates. (**B**) Distribution of Terminational Stalling Index (TSI) in Col0, *xrn4* and *fry1* and comparison of XRN4 and FRY1 targets in shoot. (**C**) Distribution of Terminational Stalling Index (TSI) in Col0, *xrn4* and *fry1* and comparison of XRN4 and FRY1 targets in roots. Only transcripts with a TSI > 3 in Col0 were kept. A transcript was considered as a XRN4 or FRY1 target when the TSI value in *xrn4* and/or *fry1* is at least two times lower than in Col0. *N* = 3 biological replicates. Significance of the distribution was tested using a Wilcoxon test. ***: p-value < 0.001

XRN exoribonuclease activity can be inhibited by 3’-phosphoadenosine 5’-phosphate (PAP) [41]. Under unstressed conditions, PAP is rapidly degraded by the FRY1 phosphatase. FRY1 loss-of-function leads to constitutive PAP accumulation and this accumulation inhibits XRN activity [39, 41, 42]. However, the detailed mechanism of this inhibition and the consequences for mRNA turnover are unclear. Here, we tested if FRY1 through PAP accumulation could be involved in CTRD modulation. First, we investigated XRN4 accumulation in polysomes (as a read-out of CTRD activity) in shoot and root of 15-d-old Col0 and *fry1* mutant seedlings (Fig. 1). After polysome profiling (Fig. 1A-B), fractions corresponding to polysomes were collected and analysed via western blotting with an XRN4-specific antibody (Fig. 1C-D). While the XRN4 signal is similar in each input fraction, its accumulation in polysomes seems to be affected in both *fry1* shoot and root. We confirmed this effect by comparing XRN4 signal in a free mRNP fraction versus a ribosome-bound fraction (Supplemental Fig. 1). To test if FRY1 inactivation impairs CTRD activity, we then performed a 5’Pseq approach in Col0, *xrn4* and *fry1* mutants (Fig. 2 and Supplemental Figs. 2–3). 5’Pseq was performed on 3 biological samples, revealing reproducible results between genotypes and organs (Supplemental Fig. 2). To assess CTRD activity, we first performed a metagene analysis around stop codons in each condition (Fig. 2A). A clear overaccumulation of 5’P reads at position − 16/-17nt before stop codons is observed in Col0 shoot and root, a hallmark of active CTRD. This peak drastically decreased in both *xrn4* and *fry1* mutants (Fig. 2A). As metagene analysis can biases the signal to higher abundance transcripts, we determined CTRD signal at the individual transcript level using the Terminational Stalling Index (TSI [19, 20, 22]),. The higher the TSI, the greater the CTRD activity. This index was determined in shoot and root respectively for Col0, *xrn4* and *fry1* mutants (Fig. 2B-C). Only transcripts with a TSI value higher than 3 in Col0 were considered as CTRD targets [20, 22]. In *xrn4* and *fry1*, the TSI distribution drastically decreases in both shoot and root. Interestingly, this decrease is significantly stronger in *fry1* than in *xrn4* in both organs. This effect was also observed when the TSI distribution is analysed independently in each biological replicate (Supplemental Fig. 3). We then compared XRN4 and FRY1 CTRD targets. To do so, we considered transcripts that present a TSI value in *fry1* and/or *xrn4* at least two times lower compared to Col0 as FRY1 and/or XRN4 CTRD targets (Fig. 2B-C). Strong overlap between XRN4 and FRY1 CTRD targets was observed in both organs with respectively 3,614 (71% and 78% of FRY1 and XRN4 tagets resectively) and 2,594 (66% and 71.7% of FRY1 and XRN4 argets resectively) common targets in shoot and root (Fig. 2B-C, Supplemental Tables 2 and 3). These data suggest that FRY1 inactivation can inhibit XRN4-CTRD activity. As CTRD is more repressed in *fry1* than in *xrn4*, these data also suggest that other enzymes may be involved in the CTRD pathway.

### PAP treatment affects XRN4 accumulation to polysomes and mRNA stability

**Fig. 3:**
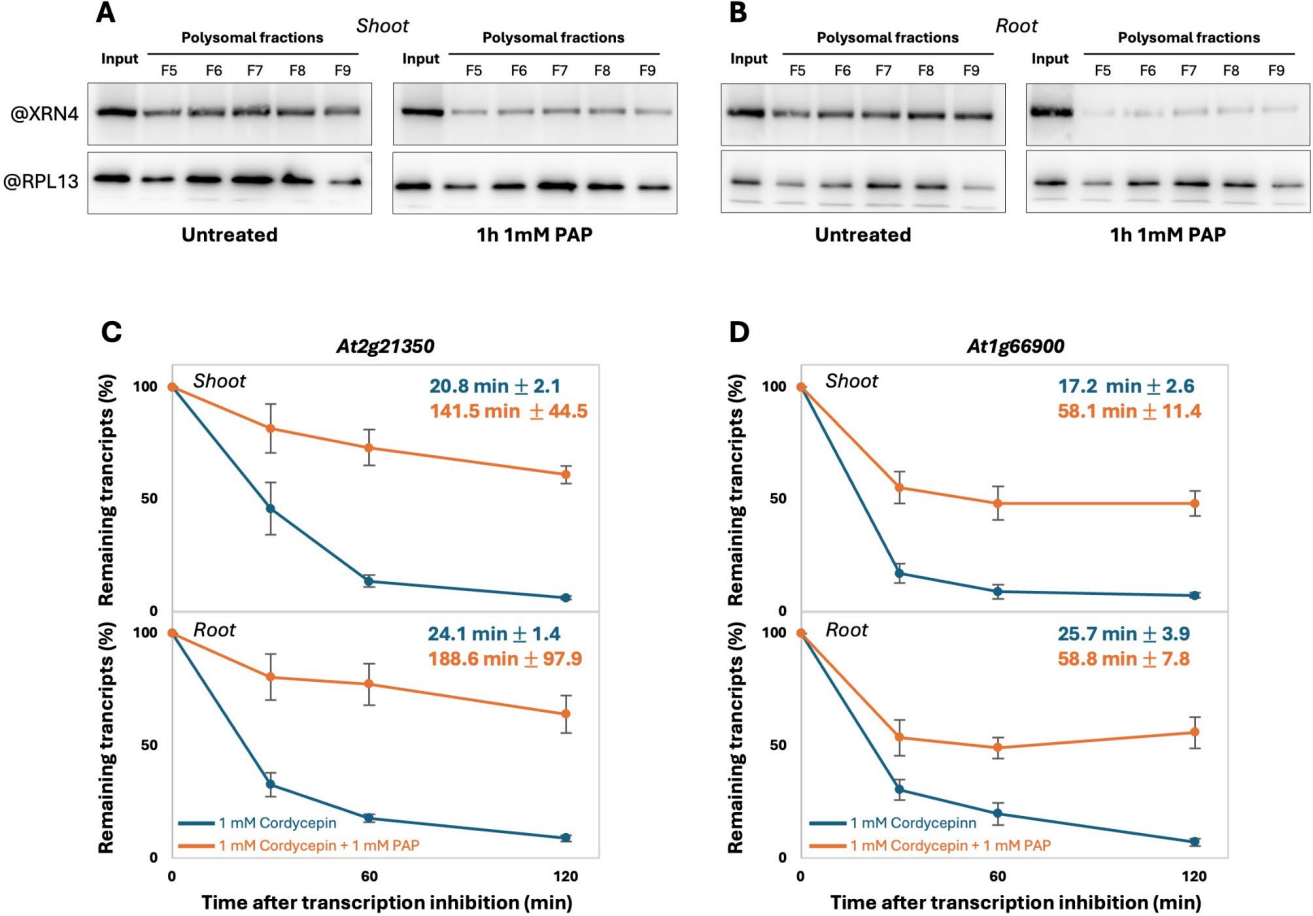
PAP treatment affects XRN4 accumulation to polysomes and mRNA stability. A. Total proteins extracted from polysomal and input fractions prepared from (**A**) Col0 shoot or (**B**) Col0 root incubated for 1 h on liquid MS medium (untreated) or 1 h on liquid MS medium supplemented with 1 mM PAP. The four blots were prepared and analysed simultaneously. The same quantities of tissues were used for each condition (e.g. 300 mg of biomass). **C**, **D**. mRNA stability was determined in vivo after 1 mM cordycepin treatment (blue line) or after 1 mM cordycepin and 1 mM PAP treatment (orange line) followed by RT-ddPCR. Half-lives are indicated on each graph. Mean ± SD. *N* = 3 biological replicates

As a constitutive accumulation of PAP is observed in *fry1* mutant [40], we tested whether exogenous PAP treatment can also affect CTRD activity. To do so, whole seedlings were treated in vivo with 1 mM exogenous PAP. Exogenous ATP was also added as is a known co-substrate for the PAP transporter [50, 52]. After 1 h of treatment, shoots and roots were collected separately. After polysome fractionation, XRN4 accumulation in polysomes was tested via western blotting (Fig. 3A-B). Interestingly, exogenous PAP treatment reduces XRN4 accumulation in polysomes in both organs without affecting XRN4 signal in input fraction. PAP treatment induces XRN4 accumulation in the free mRNP fraction (Supplemental Fig. 4). To test if PAP can affect CTRD activity, we measured the mRNA half-lives of candidate CTRD targets after cordycepin treatment supplemented with 1 mM PAP (Fig. 3C-D). We choose two transcripts targeted by CTRD that present lower TSI in *fry1* and *xrn4* mutants. For these two candidate targets, PAP treatment significantly affects mRNA stability in both organs. For At2g21350 transcript, mRNA half-life varied from 20.8 min to 141.5 min (t.test p-value < 0.05) and from 24.1 min to 188.6 min (t.test p-value < 0.05) after PAP treatment in shoot and root respectively. At1g66900 transcript followed the same trend with an mRNA half-life that varies from 17.2 min to 58.1 min (t.test p-value < 0.01) and from 25.7 min to 58.8 min (t.test p-value < 0.05) after PAP treatment in shoot and root respectively (Fig. 3C-D). However, a non XRN4/FRY1 CTRD targets is not impacted by PAP treatment in both organs (Supplemental Fig. 5, t.test p-value = non-significant). Collectively, these data suggest that PAP can regulate directly or indirectly CTRD activity in shoot and root.

### DXO1 catalytic inactivation affects co-translational mRNA decay

**Fig. 4:**
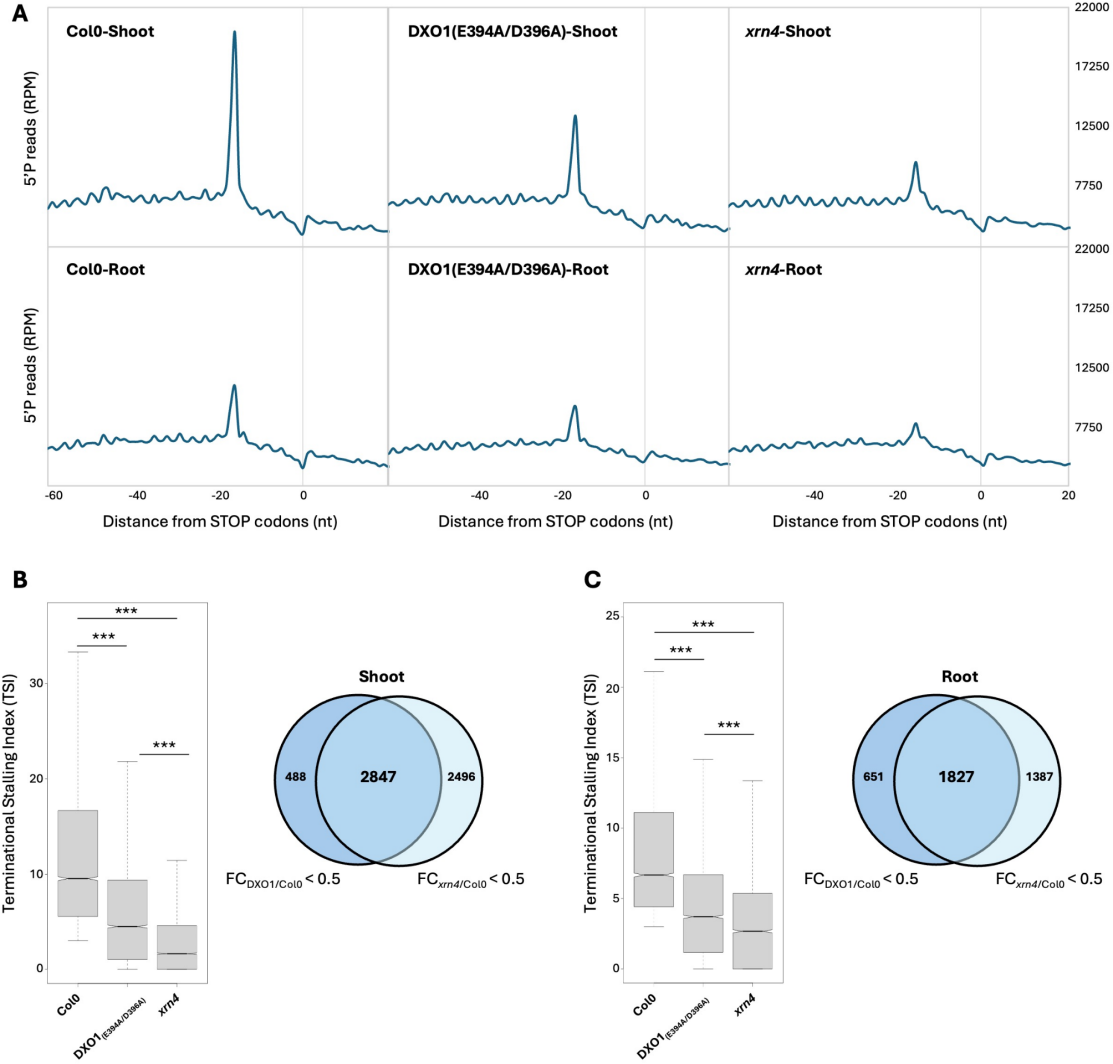
DXO1 catalytic inactivation affects co-translational mRNA decay in both shoot and root. (**A**) Metagene analysis of 5′P reads accumulation around stop codons. The different profiles are representative of 3 biological replicates. (**B**) Distribution of Terminational Stalling Index (TSI) in Col0, DXO1(E394A/D396A) and *xrn4* and comparison of XRN4 and DXO1 targets in shoot. (**C**) Distribution of Terminational Stalling Index (TSI) in in Col0, DXO1(E394A/D396A) and *xrn4* and comparison of XRN4 and DXO1 targets in root. Only transcripts with a TSI > 3 in Col0 were kept. A transcript was considered as a XRN4 or DXO1 target when the TSI value in *xrn4* and/or DXO1(E394A/D396A) is at least two times lower than in Col0. *N* = 3 biological replicates. Significance of the distribution was tested using a Wilcoxon test. ***: p-value < 0.001

Recently, it was reported that in addition to XRN activity, PAP can also inhibit in vitro DXO1 exoribonuclease activity [43]. DXO1 is implicated in the removal of non-canonical NAD^+^ cap and in RNA turnover [43, 53]. However, its contribution to CTRD has never been tested. We first tested DXO1 association with ribosomes using a complemented DXO1-GFP line [43]. As for XRN4, DXO1 distribution in free mRNP and ribosome-bound fractions was analyzed (Supplemental Fig. 6). A DXO1 signal can be detected in both fractions with a higher signal in the ribosome-bound fraction. Interestingly, as for XRN4, a PAP treatment also affects DXO1 association with ribosomes (Supplemental Fig. 6). We then tested DXO1 implication in CTRD activity. As the *dxo1* knockout mutant presents drastic phenotypes not directly linked to its catalytic activity [43, 47], we used a catalytically inactive DXO1, DXO1(E394A/D396A) expressed in a *dxo1-2* knockout mutant [43]. In comparison to Col0 and *xrn4*, we performed a 5’Pseq analysis in DXO1(E394A/D396A) shoot and root (Fig. 4). 5’Pseq was performed on 3 biological samples, revealing reproducible results between genotypes and organs (Supplemental Fig. 7). Metagene analysis reveals a decrease of the peak at position − 16/-17nt before stop codons in DXO1(E394A/D396A) line compared to Col0 but to a lesser extent than in *xrn4* in both shoot and root (Fig. 4A). To confirm this moderate effect, we analysed the TSI distribution in each genotype (Fig. 4B-C). In both organs, the TSI distribution significantly decreased in DXO1(E394A/D396A) and *xrn4* lines. Interestingly, the decrease is significantly lower in *xrn4* than in DXO1(E394A/D396A) line in shoot and in root. However, a clear overlap between the DXO1 and XRN4 CTRD targets was observed with 85.3.% and 73.7% of DXO1 targets are also XRN4 targets in shoot and root respectively (Fig. 4B-C, Supplemental Tables 4 and 5). These data suggest that DXO1 and XRN4 can share similar targets.

### DXO1 co-translational mRNA decay targets are identified as NAD ^+^-capped RNAs and targeted by FRY1

**Fig. 5:**
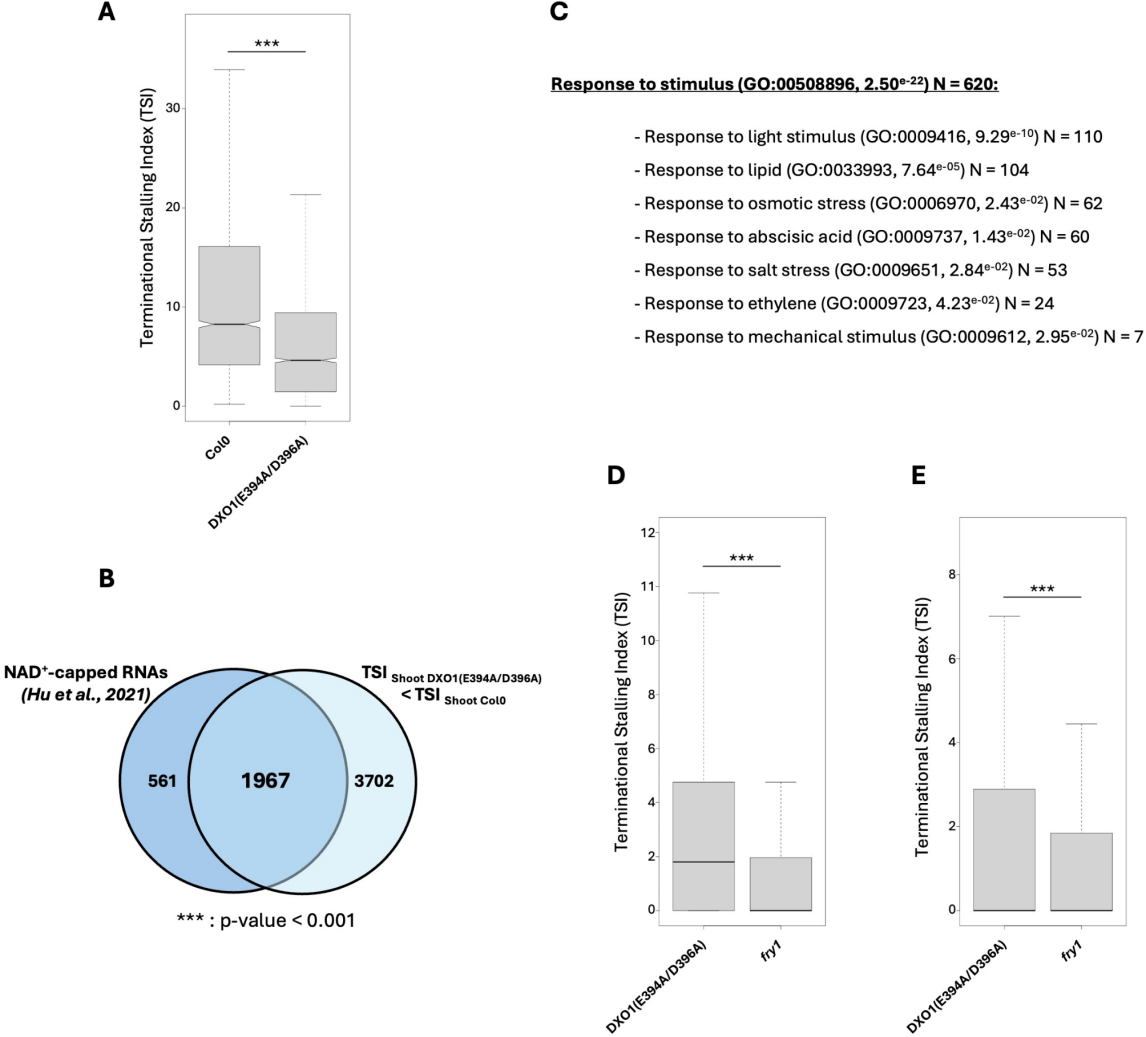
DXO1 co-translational mRNA decay targets are identified as NAD^+^-capped RNAs and targeted by FRY1. (**A**) Distribution of Terminational Stalling Index (TSI) of NAD^+^-capped RNAs (identified in Hu et al., 2021) in Col0 and DXO1(E394A/D396A) line, *N* = 3,011. Transcripts with a TSI value higher than 0 in Col0 were kept for the analysis. Significance of the distribution was tested using a Wilcoxon test. ***: p-value < 0.001. (**B**) Venn diagram representation of NAD^+^-capped transcripts targeted by CTRD (TSI > 3 in Col0) and transcripts with a lower TSI value in DXO1 (E394A/D396A) compared to Col0. To test the significance of the overlap, hypergeometric test was performed (***: p-value < 0.001). (**C**) List of response to stimulus GO terms for the overlap targets (*N* = 1,967). The number of genes per category is indicated. **D**, **E**. Distribution of the TSI for the common targets of DXO1(E394A/D396A) and FRY1 in shoot and root respectively. Significance of the distribution was tested using a Wilcoxon test. ***: p-value < 0.001

As DXO1 is known to be involved in the removal of non-canonical NAD^+^ cap, we tested whether DXO1-identified co-translational mRNA decay targets can harbour a NAD^+^ cap. Recently, a survey of NAD^+^-capped RNAs was performed using SPAAC-NAD-seq approach [48]. Thus, we compared NAD^+^-capped RNAs with DXO1 co-translational mRNA decay targets. Given that the SPAAC-NAD-seq approach was performed on seedlings, we compared the data with DXO1 CTRD targets in shoot. Moreover, as the two datasets were obtained from different developmental stages (12- and 15-d-old seedlings), we retained only the transcripts identified in both datasets resulting in a total of 3,011 transcripts harbouring a NAD^+^ cap identified in our analysis. For these transcripts, we analysed the TSI distribution in Col0 and DXO1(E394A/D396A) line (Fig. 5A). Interestingly, 83.3% of the NAD^+^ cap identified transcripts (2,511/3,011) are targeted by CTRD in Col0 (TSI > 3). Additionally, the TSI distribution is significantly lower in DXO1(E394A/D396A) than in Col0 (Fig. 5A). In fact, a significant overlap (1,957/2,586, p-value < 0.001) is observed between CTRD targets harboring a NAD^+^ cap and transcripts with a lower TSI value in DXO1(E394A/D396A) (Fig. 5B). In order to determine biological processes targeted by DXO1 co-translational mRNA decay, a Gene Ontology (GO) analysis was performed (*N* = 1,957, Fig. 5C). Among the different GO terms identified, many terms associated with “Response to stimulus” were enriched in the dataset (GO = 00508896, 2.50^e − 22^, Fig. 5C and Supplemental Table 6). Among them, the GO term “Response to abscisic acid” was significantly enriched (GO:0009737, 1.43^e − 02^). Interestingly, DXO1 has been reported to regulate mRNA stability of ABA-related NAD^+^-capped mRNAs [53]. As DXO1 is also sensitive to PAP, we compared DXO1 targets to FRY1 targets in shoot and root (Fig. 5D-E). The TSI distribution in both organs reveals a stronger effect in *fry1* than in DXO1(E394A/D396A). Taken together, these data suggest that NAD^+^ cap transcripts can be co-translationally decayed, probably by DXO1.

## Discussion

Since its discovery, the CTRD pathway has been found to globally shape the whole transcriptome in several organisms [11, 20, 22, 24, 26, 54]. However, details about its regulation are still rare in the literature. Here, using polysomes analysis, 5’Pseq approaches and mRNA half-life determination, we reported that PAP and DXO1 are involved in CTRD regulation in both shoot and root in Arabidopsis.

XRN4 was described as the main enzyme involved in CTRD in Arabidopsis [20, 21, 23, 24]. However, we and others have reported that a slight CTRD activity is still present in the *xrn4* mutant [20, 21, 23]. In fact, the 5’P reads accumulation 16/17 nucleotide before stop codons is not totally abolished in the *xrn4* mutant [20, 21]. Moreover a 3-nt periodicity is still observed in the *xrn4* mutant suggesting that additional pathways can contribute to this periodicity. Recent studies have proposed that ribosome dynamics can also be linked with endoribonuclease activity. For example, in yeast, ribosome collisions can trigger mRNA cleavage at ribosome boundaries via the endoribonucleases Cue2 and SmrB [55, 56]. In Arabidopsis, only one endoribonuclease was recently characterized [57, 58]. However, its link with ribosome dynamics has not been assessed. CTRD mediated by other exoribonucleases could also explain the remaining slight CTRD activity in the *xrn4* mutant. Arabidopsis genome encodes three XRN genes, XRN2, XRN3 and XRN4. Recently, a large overview of the Arabidopsis mRNA degradome was obtained via degradome sequencing of the different *xrn* mutants in addition to *fry1* [23]. It appears that XRN2 and XRN3 have no redundant functions with XRN4 in the CTRD. This analysis was performed only on single mutants and cannot reveal compensate mechanism involving XRN2 and/or XRN3. Additionally, this analysis revealed that mRNA decay is more repressed in *fry1* than in *xrn4* [23]. These data are consistent with our findings. In fact, through TSI analysis, our data revealed that XRN4 and FRY1 share similar targets with a stronger effect in *fry1* than in *xrn4* (Fig. 2).

To test the potential involvement of other exoribonucleases in CTRD, we tested the contribution of DXO1 to this process. In Arabidopsis, DXO1 was demonstrated to possess deNADding and exoribonuclease activities [43, 59]. DXO1 inactivation induces pleiotropic phenotypes such as growth defects, pale green coloration or insensitivity to ABA [43, 53, 59]. Our data revealed that DXO1 contributes to CTRD but to a lesser extent than XRN4 does (Fig. 4). Using published SPAAC-NAD-seq data, we also identified that DXO1 CTRD targets possess a NAD^+^ cap suggesting that NAD^+^ cap transcripts can be targeted to degradation by a co-translational decay process. This is supported by the identification of NAD^+^ transcripts in polysomes [60]. The lower contribution of DXO1 in CTRD compared to XRN4 can be explained by the fact that DXO1 is a distributive enzyme while XRN4 harbours processive activity [61]. After deNADding by DXO1, XRN4 can probably compete with DXO1 for co-translational mRNA decay. Recently, it was proposed that XRN1 in yeast can also harbour a deNADding activity [62]. But this has not yet been demonstrated for XRN4. As our approach cannot discriminate between 5’P co-translational mRNA decay intermediates coming from a canonical cap and 5’P co-translational mRNA decay intermediates coming from a NAD^+^ cap, we cannot exclude also that XRN4 and DXO1 target similar transcripts but with different caps. Finally, the moderate impact of DXO1 on global CTRD can be explained by its selectivity for only NAD^+^-capped mRNAs. Recently in Arabidopsis, the absence of XRN4-CTRD pathway in roots was proposed to result in a large feedback mechanism by an unknown decay pathway [20] suggesting that other exoribonucleases can also compensate for the absence of XRN4-CTRD.

Our data also reveal that PAP can affect CTRD activity and XRN4/DXO1 association to ribosomes. A biochemical characterization of Human XRN1 in interaction with PAP was recently performed [63]. The crystal structure reveals that PAP bounds to the active site of XRN1, the adenine base of PAP forms a π-stacking interaction with the amino acid His41 [63]. Interestingly, this amino acid appears conserved and is also important for XRN1 interaction to the ribosome in yeast and drosophila [38] suggesting that PAP can interfere the interaction XRN1/Ribosome and impaired CTRD activity. This amino acid is also present in AtXRN4 (His61) suggesting a conserved regulatory mechanism [20]. Our data reveal that DXO1 is found in polysomes. How does DXO1 interact with the ribosome is still an open question.

The GO analysis also revealed that DXO1 CTRD targets are enriched in mRNAs responsible for stress responses, particularly mRNAs linked to the ABA response (Fig. 5C). This term is consistent with the proposed role of DXO1 in ABA-related transcript stability [53]. In addition, other GO terms linked to stress response appears also enriched. Future experiments will be needed to determine the direct link between DXO1-CTRD pathway and stress response in Arabidopsis.

## Conclusions

In conclusion, our study revealed that the CTRD pathway can be modulated by PAP and triggered by DXO1 in Arabidopsis. For the first time, we demonstrated the role of another exoribonuclease, DXO1, in the regulation of this pathway, probably by targeting NAD^+^-capped mRNAs. Finally, this study paves the way for studying the regulatory importance of CTRD in plants.

## Electronic supplementary material

Below is the link to the electronic supplementary material.

Supplementary Material 1: **Supplemental Fig.1**: XRN4 is accumulated in free mRNP fraction in fry1 mutant. Distribution of XRN4 in free mRNP (Pool of F1 to F3) and ribosome-bound fractions (Pool of F4 to F9) in Col0 and fry1 using XRN4 specific antibody. The same percentage of each pool was loaded. UGPase antibody was used as a negative control

Supplementary Material 2: **Supplemental Fig.2**: Principal component analysis for Col0, xrn4 and fry1 5’Pseq samples. A. Shoot samples. B. Root samples. N = 3 biological replicates per genotype per organ.

Supplementary Material 3: **Supplemental Fig.3**: Distribution of Terminational Stalling Index (TSI) in Col0, xrn4 and fry1 in each individual replicate. A-C. Distribution in shoot replicates. D-F. Distribution in root replicates. Significance of the distribution was tested using a Wilcoxon test. ***: p-value  < 0.001.

Supplementary Material 4: **Supplemental Fig.4**: XRN4 is accumulated in free mRNP fraction upon PAP treatment. Distribution of XRN4 in free mRNP (Pool of F1 to F3) and ribosome-bound (Pool of F4 to F9) fractions in Col0 shoots incubated for 2 hours on liquid MS medium (untreated) or 2 hours on liquid MS medium supplemented with 1 mM PAP. The same percentage of each fraction was loaded. UGPase antibody was used as a negative control.

Supplementary Material 5: **Supplemental Fig.5**: PAP treatment did not affect mRNA stability of a non-XRN4/FRY1 target. mRNA stability was determined in vivo after 1 mM cordycepin treatment (blue line) or after 1 mM cordycepin and 1 mM PAP treatment (orange line) followed by RT-ddPCR. Half-lives are indicated on each graph. Mean ± SD. N = 3 biological replicates.

Supplementary Material 6: **Supplemental Fig.6**: DXO1 is accumulated in polysomes and sensitive to PAP treatment. Distribution of DXO1 in free mRNP (Pool of F1 to F3) and ribosome-bound (Pool of F4 to F9) fractions in DXO1-GFP line incubated for 2 hours on liquid MS medium (untreated) or 2 hours on liquid MS medium supplemented with 1 mM PAP. The same percentage of each fraction was loaded. GFP antibody was used to detect GFP-DXO1 signal. UGPase antibody was used as a negative control.

Supplementary Material 7: **Supplemental Fig.7**: Principal component analysis for Col0, xrn4 and DXO1(E394A/D396A) 5’Pseq samples. A. Shoot samples. B. Root samples. N = 3 biological replicates per genotype per organ.

Supplementary Material 8: **Supplemental Fig.8**: Distribution of Terminational Stalling Index (TSI) in Col0, DXO1(E394A/D396A) and xrn4 in each individual replicate. A-C. Distribution in shoot replicates. D-F. Distribution in root replicates. Significance of the distribution was tested using a Wilcoxon test. ***: p-value < 0.001.

Supplementary Material 9: **Supplemental Table 1**. List of primers using for RT-ddPCR.

Supplementary Material 10: **Supplemental Table 2**. List of genes identified as co-translational mRNA decay targets in shoot and targeted by XRN4 and/or FRY1.

Supplementary Material 11: **Supplemental Table 3**. List of genes identified as co-translational mRNA decay targets in root and targeted by XRN4 and/or FRY1.

Supplementary Material 12: **Supplemental Table 4**. List of genes identified as co-translational mRNA decay targets in shoot and targeted by DXO1 and/or XRN4.

Supplementary Material 13: **Supplemental Table 5**. List of genes identified as co-translational mRNA decay targets in root and targeted by DXO1 and/or XRN4.

Supplementary Material 14: **Supplemental Table 6**. GO terms enrichment analysis.

## Data Availability

The 5’Pseq datasets generated in this study have been deposited in the Short Read Archive with the accession code PRJNA1185437 and PRJNA1189285.

## Abbreviations

CTRD: Co-Translational mRNA Decay
DXO1: Decapping and exoribonuclease protein 1
FRY1: FIERY1
NAD: Nicotinamide Adenine Dinucleotide
PAP: 3ʹ-PhosphoAdenosine 5ʹ-Phosphate
XRN4: Exoribonuclease 4
