# Supplementary figures and images for: Regulation of co-translational mRNA decay by PAP and DXO1 in Arabidopsis
Source: BMC Plant Biol. 2025 Feb 18;25:223. doi: 10.1186/s12870-025-06195-5 (PMC11834196; doi:10.1186/s12870-025-06195-5)

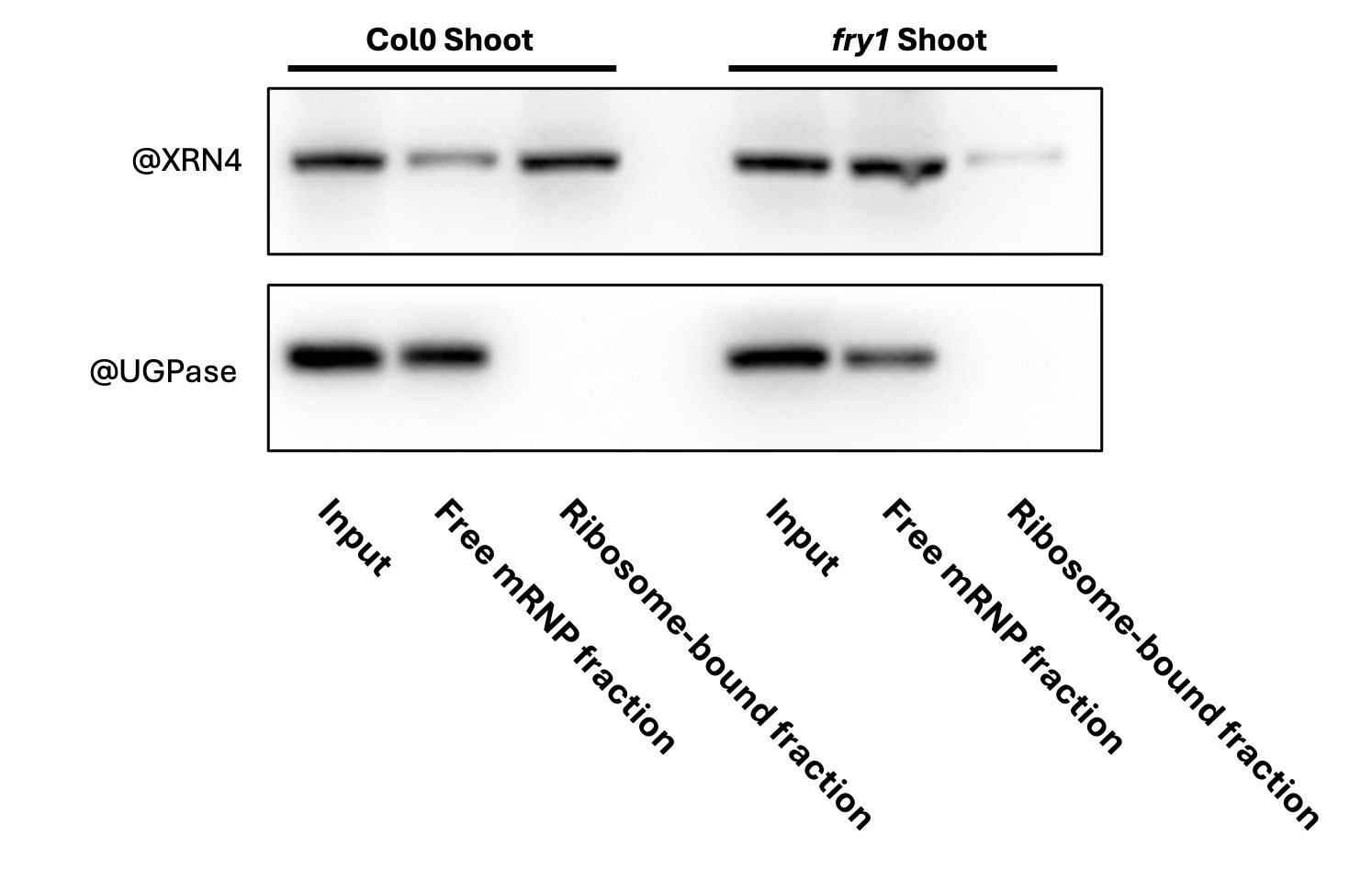

Supplement: Supplementary file 1 — Supplementary Material 1: Supplemental Fig.1: XRN4 is accumulated in free mRNP fraction in fry1 mutant. Distribution of XRN4 in free mRNP (Pool of F1 to F3) and ribosome-bound fractions (Pool of F4 to F9) in Col0 and fry1 using XRN4 specific antibody. The same percentage of each pool was loaded. UGPase antibody was used as a negative control [file 12870_2025_6195_MOESM1_ESM.jpg]

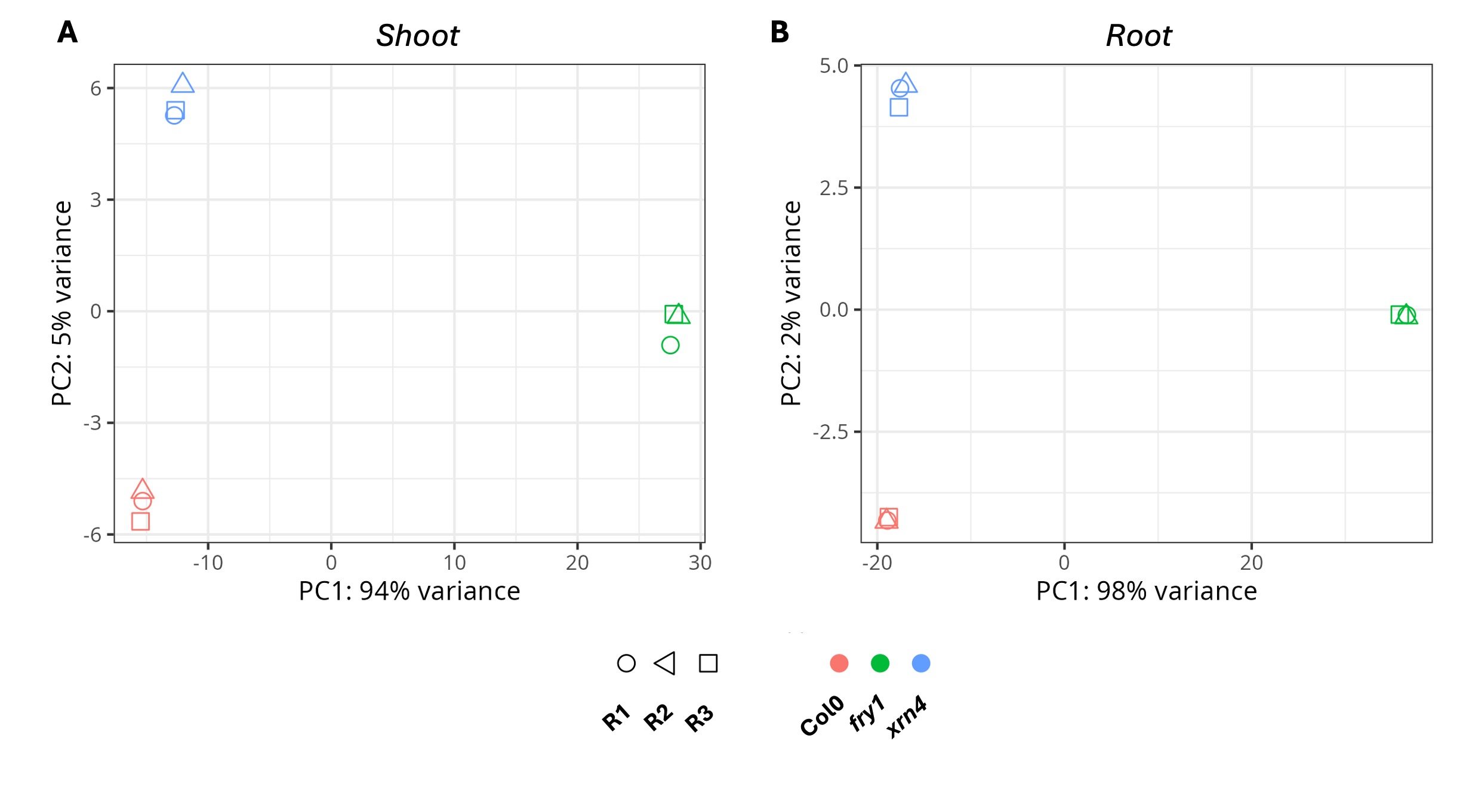

Supplement: Supplementary file 2 — Supplementary Material 2: Supplemental Fig.2: Principal component analysis for Col0, xrn4 and fry1 5’Pseq samples. A. Shoot samples. B. Root samples. N = 3 biological replicates per genotype per organ. [file 12870_2025_6195_MOESM2_ESM.jpg]

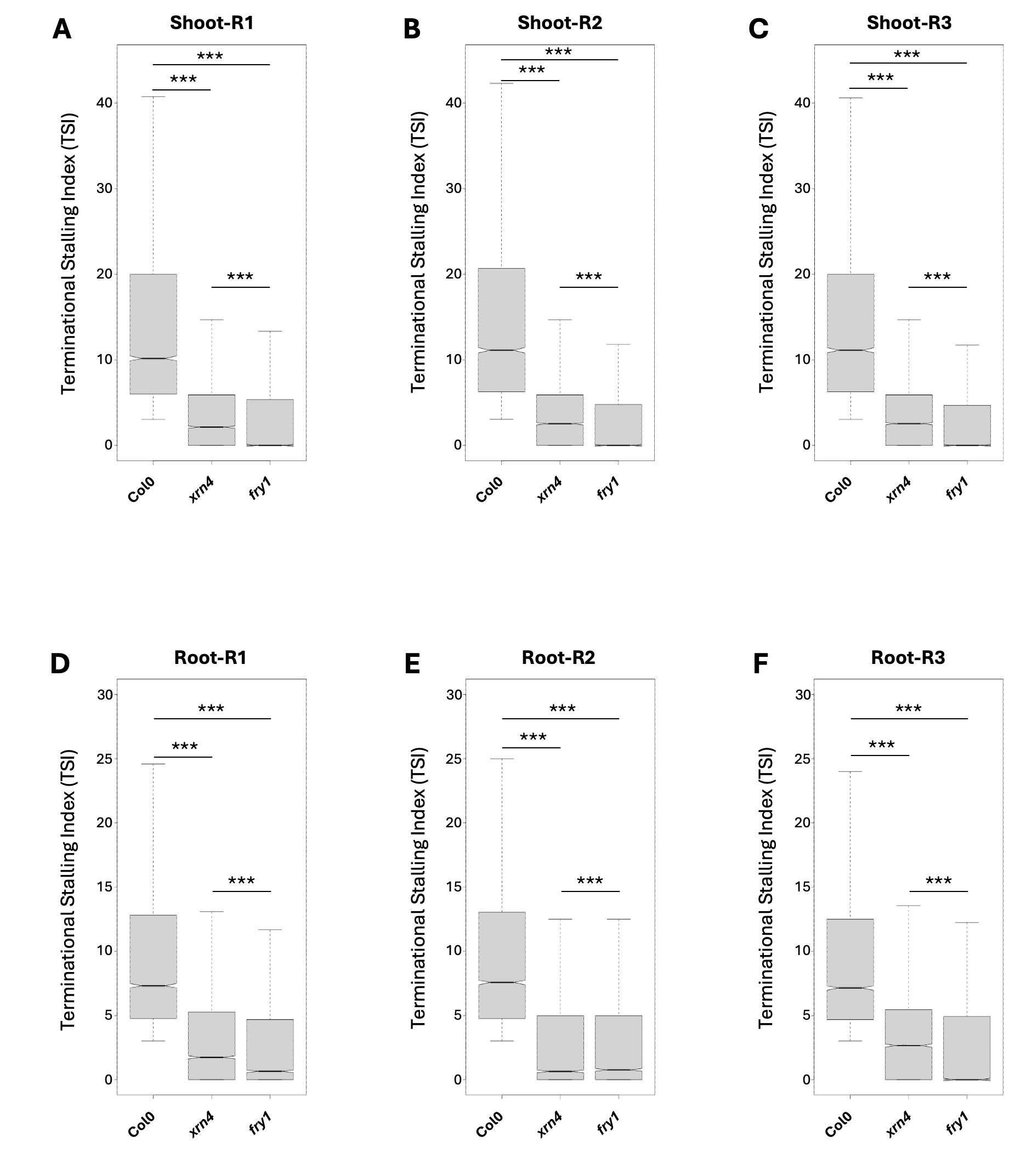

Supplement: Supplementary file 3 — Supplementary Material 3: Supplemental Fig.3: Distribution of Terminational Stalling Index (TSI) in Col0, xrn4 and fry1 in each individual replicate. A-C. Distribution in shoot replicates. D-F. Distribution in root replicates. Significance of the distribution was tested using a Wilcoxon test. ***: p-value < 0.001. [file 12870_2025_6195_MOESM3_ESM.jpg]

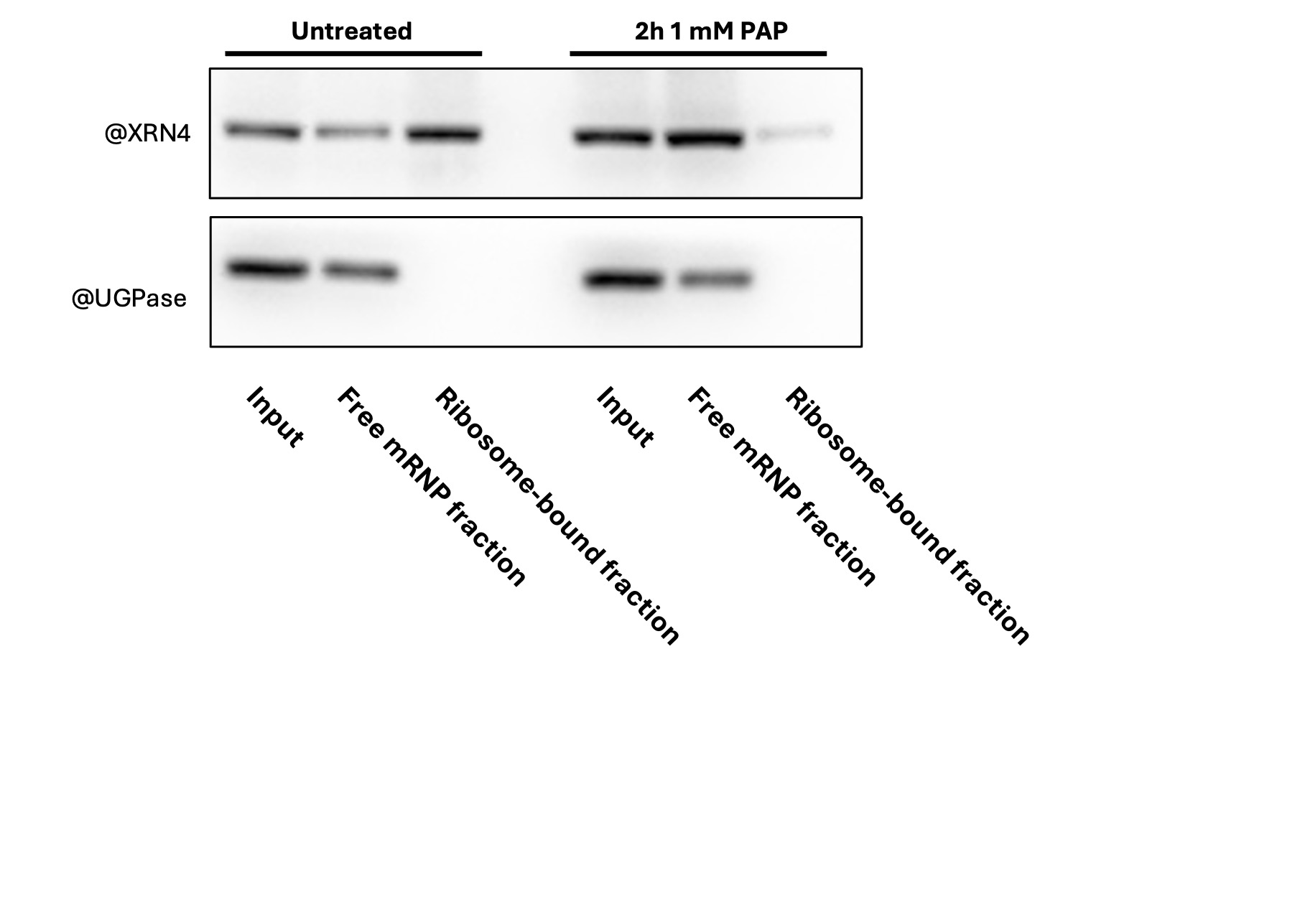

Supplement: Supplementary file 4 — Supplementary Material 4: Supplemental Fig.4: XRN4 is accumulated in free mRNP fraction upon PAP treatment. Distribution of XRN4 in free mRNP (Pool of F1 to F3) and ribosome-bound (Pool of F4 to F9) fractions in Col0 shoots incubated for 2 hours on liquid MS medium (untreated) or 2 hours on liquid MS medium supplemented with 1 mM PAP. The same percentage of each fraction was loaded. UGPase antibody was used as a negative control. [file 12870_2025_6195_MOESM4_ESM.jpg]

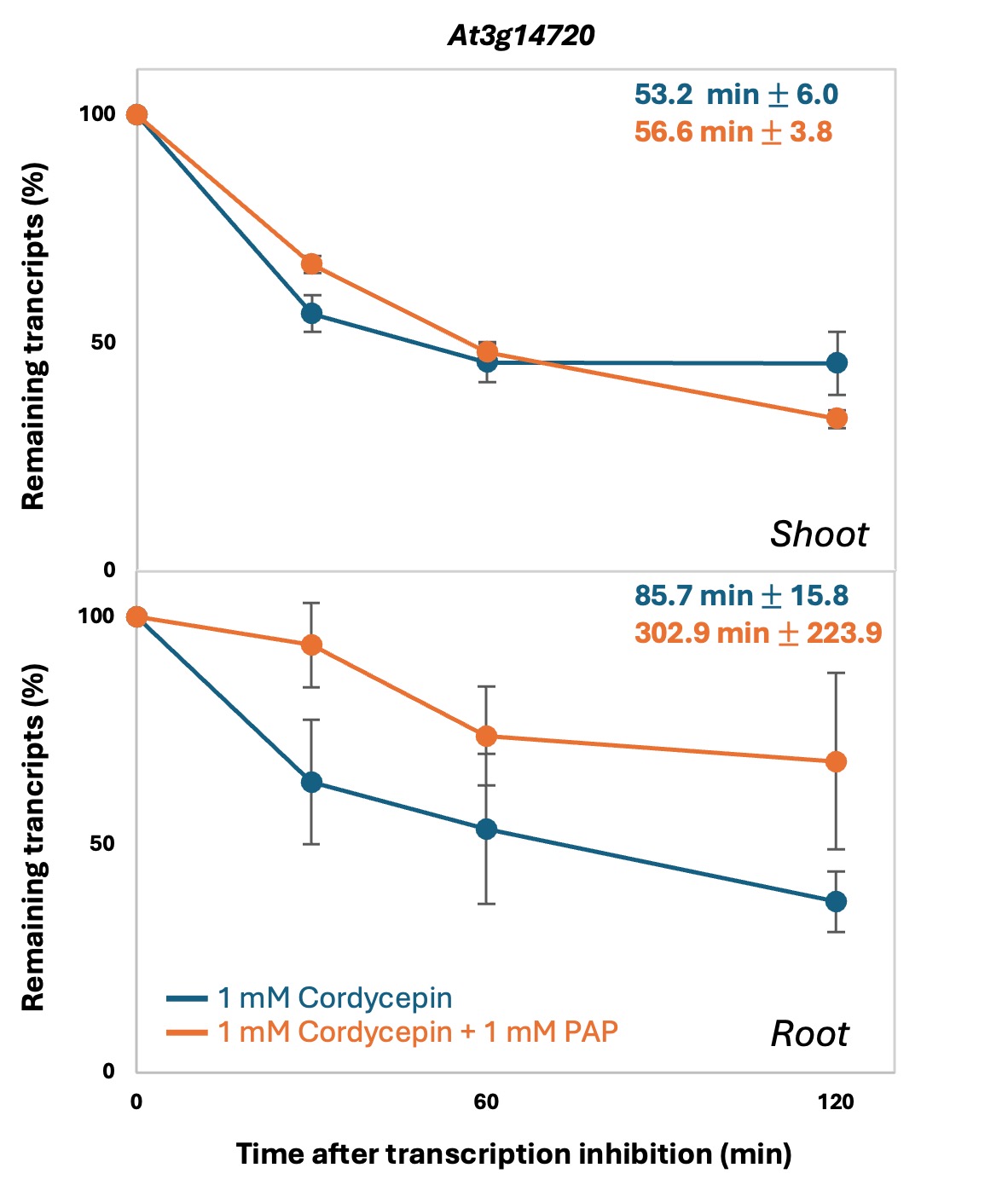

Supplement: Supplementary file 5 — Supplementary Material 5: Supplemental Fig.5: PAP treatment did not affect mRNA stability of a non-XRN4/FRY1 target. mRNA stability was determined in vivo after 1 mM cordycepin treatment (blue line) or after 1 mM cordycepin and 1 mM PAP treatment (orange line) followed by RT-ddPCR. Half-lives are indicated on each graph. Mean ± SD. N = 3 biological replicates. [file 12870_2025_6195_MOESM5_ESM.jpg]

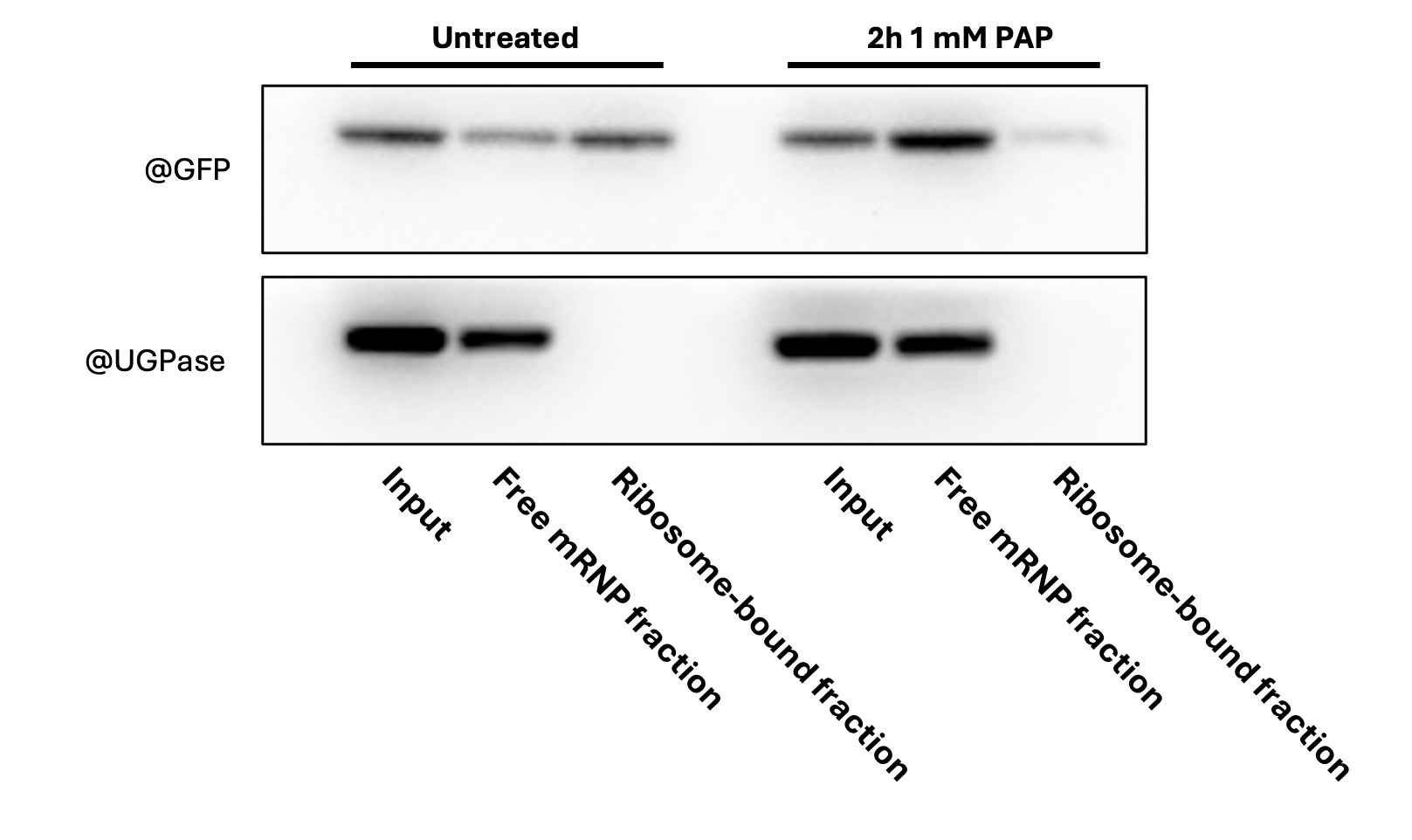

Supplement: Supplementary file 6 — Supplementary Material 6: Supplemental Fig.6: DXO1 is accumulated in polysomes and sensitive to PAP treatment. Distribution of DXO1 in free mRNP (Pool of F1 to F3) and ribosome-bound (Pool of F4 to F9) fractions in DXO1-GFP line incubated for 2 hours on liquid MS medium (untreated) or 2 hours on liquid MS medium supplemented with 1 mM PAP. The same percentage of each fraction was loaded. GFP antibody was used to detect GFP-DXO1 signal. UGPase antibody was used as a negative control. [file 12870_2025_6195_MOESM6_ESM.jpg]

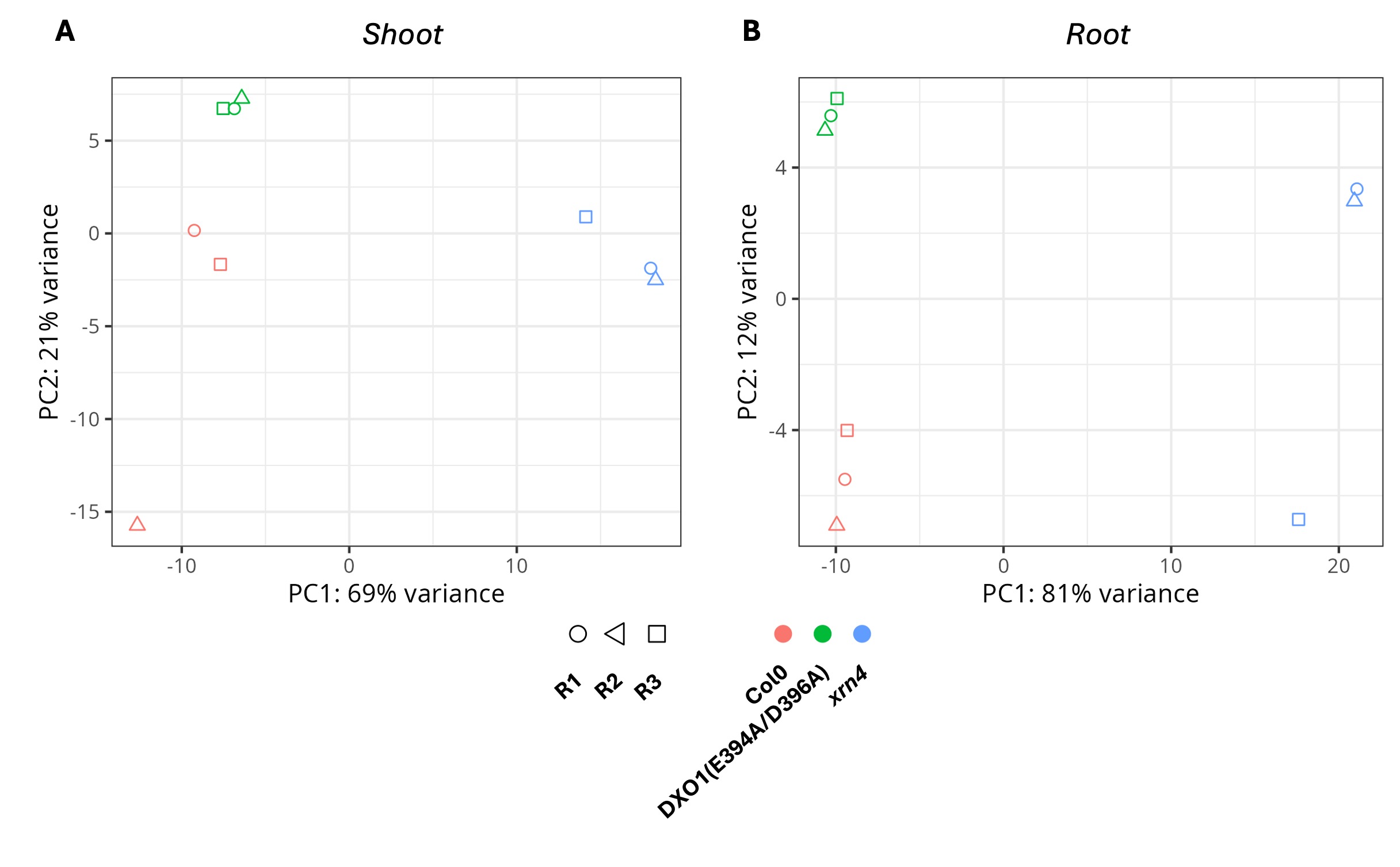

Supplement: Supplementary file 7 — Supplementary Material 7: Supplemental Fig.7: Principal component analysis for Col0, xrn4 and DXO1(E394A/D396A) 5’Pseq samples. A. Shoot samples. B. Root samples. N = 3 biological replicates per genotype per organ. [file 12870_2025_6195_MOESM7_ESM.jpg]

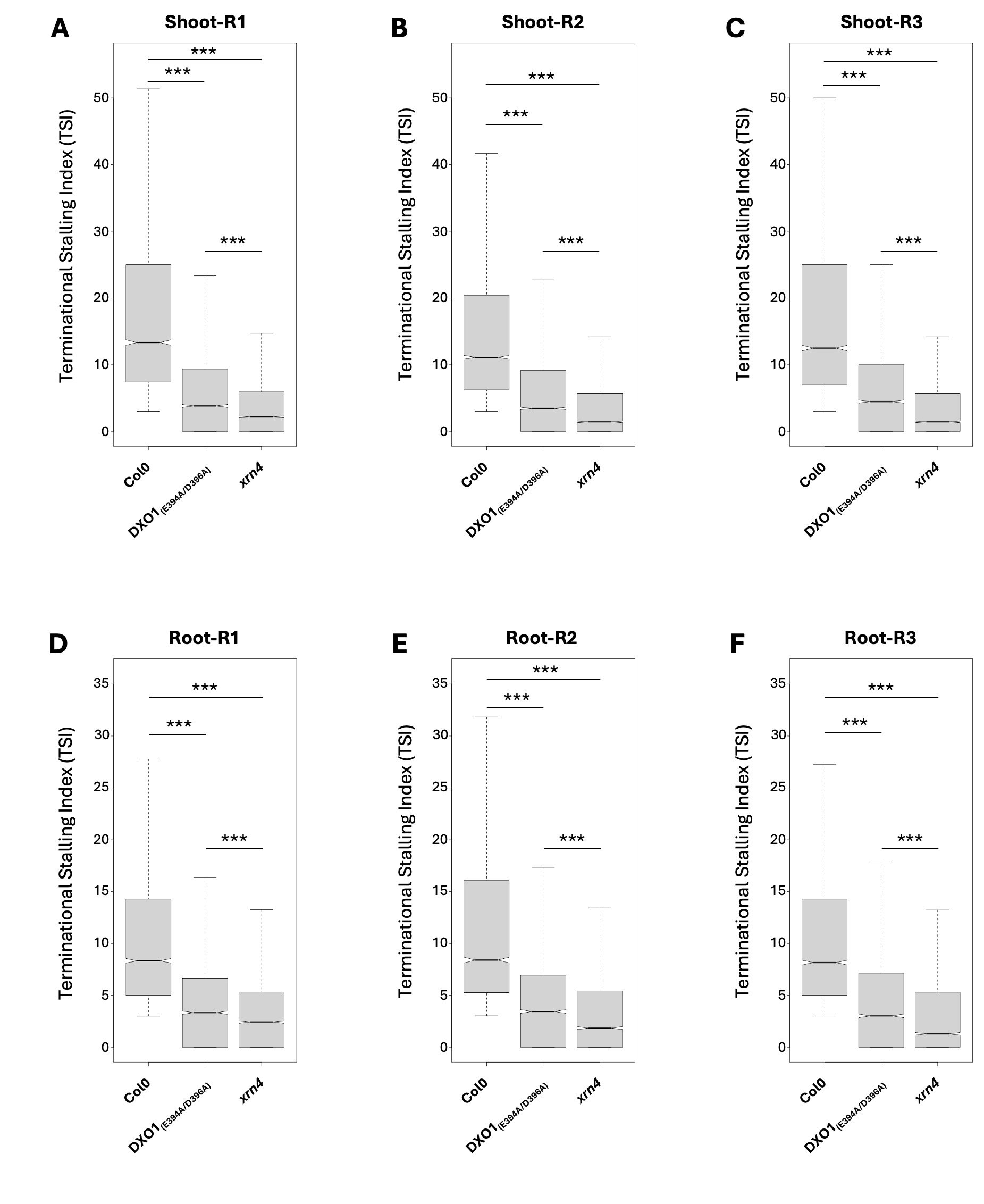

Supplement: Supplementary file 8 — Supplementary Material 8: Supplemental Fig.8: Distribution of Terminational Stalling Index (TSI) in Col0, DXO1(E394A/D396A) and xrn4 in each individual replicate. A-C. Distribution in shoot replicates. D-F. Distribution in root replicates. Significance of the distribution was tested using a Wilcoxon test. ***: p-value < 0.001. [file 12870_2025_6195_MOESM8_ESM.jpg]
